# Bio‐Based Thermoplastic Room Temperature Phosphorescent Materials with Closed‐Loop Recyclability

**DOI:** 10.1002/advs.202414439

**Published:** 2025-03-14

**Authors:** Yuanyuan Qian, Yingxiang Zhai, Meng Li, Yinping Qin, Liang Lv, Tony D. James, Lidong Wang, Zhijun Chen

**Affiliations:** ^1^ Hebei Key Lab of Power Plant Flue Gas Multi‐Pollutants Control, Department of Environmental Science and Engineering North China Electric Power University Baoding 071003 P. R. China; ^2^ Key Laboratory of Bio‐based Material Science & Technology Northeast Forestry University Harbin 150040 P. R. China; ^3^ Department of Chemistry University of Bath Bath BA2 7AY UK; ^4^ School of Chemistry and Chemical Engineering Henan Normal University Xinxiang 453007 P. R. China

**Keywords:** bio‐based polymer, closed‐loop recyclability, room‐temperature phosphorescence, thermoplastic processability

## Abstract

Producing thermoplastic room temperature phosphorescent (RTP) materials with closed‐loop recyclability from natural sources is an attractive approach but hard to achieve. Here, the study develops such RTP materials, Poly(TA)/Cell, by thermally polymerizing thioctic acid in the presence of cellulose. Specifically, polymerized thioctic acid poly(TA) forms strong hydrogen bonding interactions with CNF, promoting formation of molecular clusters between the oxygen‐containing units. The as‐formed clusters generate humidity‐ and excitation‐sensitive green RTP emission. Red afterglow emission is also obtained by integrating Poly(TA)/Cell together with Rhodamine B (RhB) via an energy transfer process. Attributed to the thermoplastic properties, the as‐obtained Poly(TA)/Cell can be thermally molded into flexible shapes with uncompromised RTP performance. Moreover, owing to the alkali‐cleavable properties of the disulfide bond in Poly(TA)/Cell, thioctic acid and cellulose can be successfully recycled from Poly(TA)/Cell with a yield of 92.3% and 81.5%, respectively. As a demonstrator for potential utility, Poly(TA)/Cell is used to fabricate materials for information encryption.

## Graphical abstract

Using the intermolecular hydrogen bonding interactions between thioctic acid and cellulose, the RTP material Poly(TA)/Cell, is developed through thermal polymerization. Attributed to the thermoplastic properties, the as‐obtained Poly(TA)/Cell can be thermally molded into flexible shapes with uncompromised RTP performance. Moreover, the monomers used for generating Poly(TA)/Cell can be efficiently recycled, as a result of the dynamic properties of the disulfide bonds.

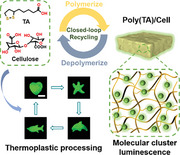

## 1 Introduction

Organic room temperature phosphorescent (RTP) materials have exhibited great potential in various applications such as for organic light‐emitting diodes,^[^ ^1^, ^2^ ^]^ biological imaging,^[^ ^3^, ^4^ ^]^ sensing,^[^ ^5^ ^]^ X‐ray scintillator,^[^ ^6^, ^7^ ^]^ passive radiative cooling,^[^ ^8^ ^]^ anti‐counterfeiting applications,^[^ ^9^, ^10^ ^]^ and information encryption^[^ ^11^, ^12^ ^]^ because of their inherent structural flexibility, tunable optical performance and relatively mild preparation.^[^ ^13^, ^14^ ^]^ Generally, two principles should be followed for designing organic RTP materials: 1) the spin‐orbit coupling (SOC) of organic chromophores should be promoted so that more triplet excitons are generated via intersystem crossing (ISC) processes. 2) radiative migration of triplet excitons to the ground state should be facilitated.^[^ ^15^ ^]^ Following these principles, numerous RTP materials including organic crystals,^[^ ^16^, ^17^, ^18^ ^]^ supramolecules,^[^ ^19^, ^20^ ^]^ polymer composites,^[^ ^21^, ^22^, ^23^ ^]^ covalent organic framework (COF),^[^ ^24^, ^25^ ^]^ metal organic framework (MOF),^[^ ^26^, ^27^ ^]^ and hydrogen‐bonded organic framework (HOF)^[^ ^28^, ^29^ ^]^ have been developed.

Recently, using renewable natural resources to produce RTP materials as a replacement for petrol‐derived resources has attracted much attention.^[^ ^30^ ^]^ This is because natural resources in general are renewable, cheap, and abundant.^[^ ^31^ ^]^ Thus, using natural resources for producing RTP materials greatly promotes the sustainability of RTP materials while also being beneficial for their practical applications. Until now, wood,^[^ ^32^, ^33^ ^]^ cellulose,^[^ ^34^, ^35^ ^]^ hemicellulose,^[^ ^36^, ^37^ ^]^ lignin,^[^ ^38^, ^39^ ^]^ gelatin,^[^ ^40^ ^]^ and natural phenolics^[^ ^41^, ^42^ ^]^ have been used for producing sustainable RTP materials. Among these natural materials with oxygen functionality, cellulose is the most abundant sustainable polymer in nature.^[^ ^43^ ^]^ It demonstrates unique advantages such as high mechanical strength, good renewability, low cost and biocompatibility.^[^ ^44^ ^]^ The rigid environment provided by cellulose molecules and multiple intra‐ and intermolecular hydrogen bonding interactions not only effectively stabilize triplet excitons and protect them from quenching but also induce them to form closer oxygen clusters for luminescence.^[^ ^45^ ^]^

Nevertheless, the as‐obtained RTP materials could not be recycled at the molecular level, compromising their sustainability. Moreover, most of these sustainable RTP materials can only be processed using solvent and lack thermoplasticity, hindering their practical applications. Poly(TA) as a novel kind of high‐performance dynamic polymer has been widely explored in different fields in recent years, such as adhesives,^[^ ^46^ ^]^ smart emissive materials,^[^ ^47^ ^]^ and sensors^[^ ^48^ ^]^ due to its self‐healing, self‐adhesive, stretchable and recyclability.^[^ ^49^ ^]^ Some pioneering groups have constructed similar materials and studied the properties of adhesion,^[^ ^50^ ^]^ self‐healing,^[^ ^51^, ^52^, ^53^ ^]^ and conductivity.^[^ ^54^, ^55^ ^]^ However, most of them ignored the interesting optical performance of such formula.

**Figure 1:**
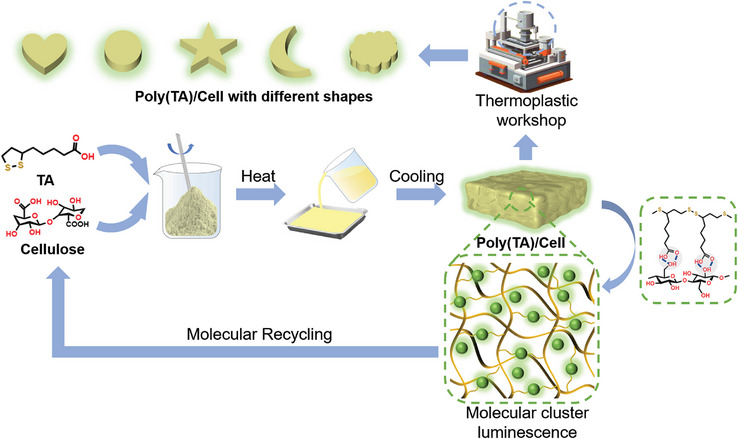
Schematic illustration for the processing and recycling of Poly(TA)/Cell with RTP.

Motivated by these challenges, we developed a closed‐loop molecular‐level recyclable and thermoplastic RTP material by polymerizing renewable thioctic acid in the presence of carboxylated cellulose nanofibers (CNF). The abundant surface hydroxyl and carboxyl groups of CNF formed intensive hydrogen bonding interactions with the polymerized thioctic acid in Poly(TA)/Cell,^[^ ^56^, ^57^ ^]^ suppressing the non‐radiative decay of triplet excitons, resulting enhanced afterglow emission lasting 186.3 ms. Additionally, Poly(TA)/Cell exhibits thermoplasticity, and as such could be processed into flexible shapes after heating. Moreover, owing to the reversible nature of the disulfide bond in Poly(TA)/Cell, this material can achieve molecular‐level closed‐loop recycling^[^ ^58^, ^59^ ^]^ through the process of depolymerization using alkaline solutions followed by acidification (**Figure** **1**).

## 2 Results and Discussion

### 2.1 Preparation and RTP of Poly(TA)/Cell

**Figure 2:**
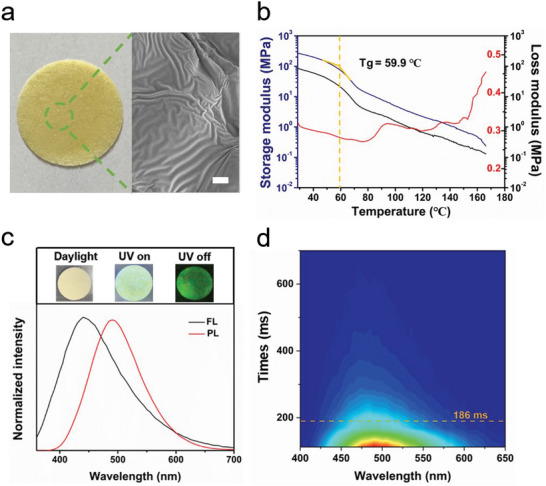
Preparation and RTP emission of Poly(TA)/Cell. a) Images of Poly(TA)/Cell (left) and scanning electron microscopic (SEM) images of Poly(TA)/Cell (right); scale bar = 5 µm. b) DMA measurement of the Poly(TA)/Cell. c) Fluorescence (FL.) and phosphorescence (PL.) emission of Poly(TA)/Cell, excitation wavelength = 300 nm, inset: images of bright field, fluorescent and RTP emission of Poly(TA)/Cell upon UV irradiation (365 nm). d) Time‐resolved RTP emission of Poly(TA)/Cell, excitation wavelength = 300 nm.

Poly(TA)/Cell was readily synthesized through a simple one‐pot reaction, where thioctic acid underwent thermally induced polymerization in the presence of CNF, resulting in a yellowish material with a green afterglow emission. SEM images showed that the CNF were well dispersed in the poly(TA) network. SEM images also indicated that the obtained Poly(TA)/Cell exhibited a wrinkled morphology, attributed to the interface instability resulting from the unstable thermal expansion ratio of the binary mixture during the heat treatment process and the cooling process (**Figure** **2**a; Figure S1, Supporting Information).^[^ ^60^, ^61^ ^]^ Subsequently, the mechanical properties of Poly(TA)/Cell were investigated using dynamic mechanical analysis (DMA). The glass transition temperature (Tg) of the Poly(TA)/Cell was determined to be 59.9 °C, and it transitions to a rubbery state at higher temperatures, indicating that Poly(TA)/Cell could be thermoplastically processed when the temperature went above 60 °C (Figure 2b). The TGA indicated that the Poly(TA)/Cell polymer maintained structural stability up to 231.3 °C (Figure S2, Supporting Information).

Subsequently, the optical performance of Poly(TA)/Cell was evaluated. Under ultraviolet (UV) irradiation, Poly(TA)/Cell exhibits strong fluorescence emission at 425 nm (Figure 2c), and green afterglow phosphorescence (RTP) emission at 500 nm was surprisingly observed after ceasing excitation, with the quantum yield was ≈1.6%. Time‐resolved spectroscopy indicated that Poly(TA)/Cell has a persistent and stable afterglow emission, with a weak phosphorescent emission lasting up to 600 ms at the same emission wavelength (Figure 2d).

### 2.2 Tunable RTP Emission of Poly(TA)/Cell

**Figure 3:**
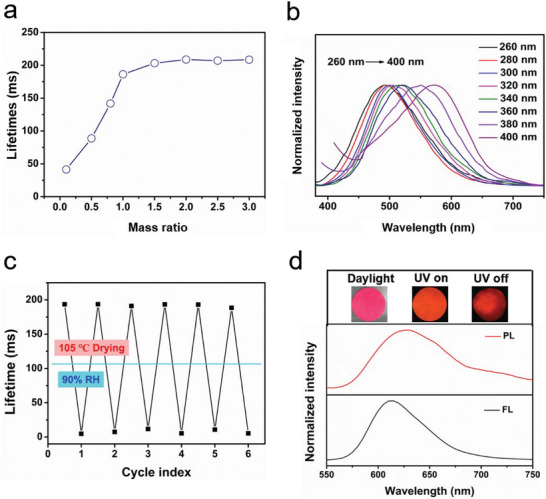
Tunable RTP emission of Poly(TA)/Cell. a) RTP lifetime of Poly(TA)/Cell with different ratio of thioctic acid and CNF. b) Normalized RTP emissions of Poly(TA)/Cell using different excitation wavelengths. c) RTP lifetimes of Poly(TA)/Cell after “drying‐humidity” cycles. d) Fluorescence and RTP spectra of Poly(TA)/Cell/RhB.

The lifetime of Poly(TA)/Cell could be adjusted by changing the mass ratio of CNF and thioctic acid. When the mass ratio of thioctic acid to CNF increased from 1:0.1 to 1:2, the RTP lifetime increased from 41.4 to 208.8 ms (**Figure** **3**a). Further increases in CNF content did not result in significant changes in the RTP intensity or lifetime of the resulting Poly(TA)/Cell (Figure S3, Supporting Information). Notably, the component materials, CNF and poly(TA), exhibited relatively shorter lifetimes of 77.0 and 112.0 ms, respectively (Figures S4 and S5, Supporting Information).

Also, the RTP emission of Poly(TA)/Cell is sensitive to the excitation wavelength. As the excitation wavelength increases from 260 to 400 nm the RTP emission of Poly(TA)/Cell exhibited an excitation‐dependent emission (Figure 3b). However, by increasing the excitation wavelengths, the emission lifetimes of the Poly(TA)/Cell tends to increase first and then decrease. When excited at 300 nm, the lifetime is at its maximum (Figure S6, Supporting Information). Additionally, Poly(TA)/Cell shows humidity‐sensitive RTP emission. As the humidity increases the RTP lifetime decreases accordingly (Figure S7, Supporting Information). When the humidity increases from 10% to 90%, the lifetime decreases from 188.4 to 4.7 ms. The results indicated that the lifetime of Poly(TA)/Cell was completely quenched when the humidity reached 100% (Figure S8, Supporting Information). After drying at high temperature, the RTP lifetime of Poly(TA)/Cell can be restored. The stability of the RTP lifetime was confirmed, where for five “wet‐dry” cycles, the RTP lifetime could be recovered, only a modest degradation in the RTP lifetime of the dried Poly(TA)/Cell was observed over the course of repeated “humidifying‐dehumidifying” cycles, confirming the robustness of RTP emission of Poly(TA)/Cell (Figure 3c). However, the problem can be solved by coating the Poly(TA)/Cell with a hydrophobic wax. After coating with wood wax, the lifetime of Poly(TA)/Cell did not decrease upon changes of humidity (Figure S9, Supporting Information).

Given that Poly(TA)/Cell could coordinate with metal cations, the RTP performance of Poly(TA)/Cell was tuned by addition of metal cations including Fe^3+^, Cu^2+^, Co^2+^, Hg^2+^, Zn^2+^, Cr^3+^, Mn^2+^, Pb^2+^, and Ca^2+^. Notably, the change of RTP performance was dependent on the types of the metal ions, indicating the potential of Poly(TA)/Cell as a sensor for these cations (Figures S10 and S11, Supporting Information).

Interestingly, the absorbance spectrum of RhB overlaps with the RTP emission spectrum of Poly(TA)/Cell, suggesting possible energy transfer between RhB and Poly(TA)/Cell (Figure S12, Supporting Information). Thus, inspired by this, the optical behavior of Poly(TA)/Cell/RhB was evaluated. As expected, Poly(TA)/Cell/RhB exhibited similar emission wavelengths in both fluorescence and phosphorescence spectra (Figure 3d). The RTP lifetime of Poly(TA)/Cell/RhB was ≈16.8 ms (Figure S13, Supporting Information). Furthermore, the delayed fluorescence and RTP characteristics of Poly(TA/Cell) after loading different amounts of RhB were investigated. With increasing RhB loading, the RTP intensity and lifetime of the Poly(TA)/Cell/RhB decreased at 490 nm and increased at 610 nm, indicating triplet‐to‐singlet Förster resonance energy transfer (TS‐FRET) between Poly(TA)/Cell and RhB was responsible for the afterglow red emission (Figures S14 and S15, Supporting Information).^[^ ^62^ ^]^

### 2.3 Mechanistic Study

**Figure 4:**
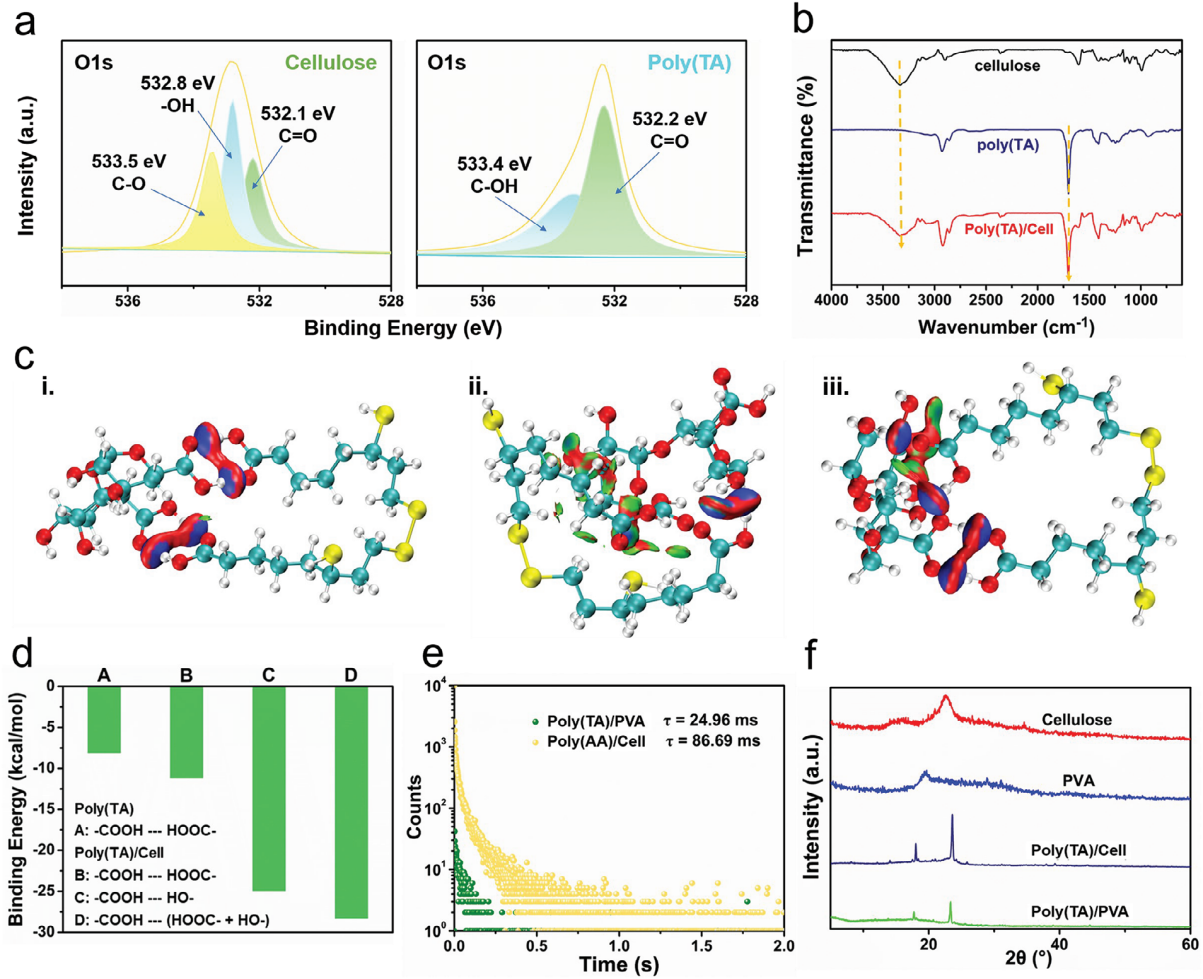
Mechanism for the RTP of Poly(TA)/Cell. a) XPS spectra of O 1s for the CNF and poly(TA). b) FTIR spectra of CNF, poly(TA) and Poly(TA)/Cell. c) A computational model for the interaction of different functional groups of Poly(TA)/Cell. d) Binding energy of Poly(TA)/Cell. e) RTP decay profiles of Poly(TA)/PVA and Poly(AA)/Cell. f) XRD patterns of CNF, PVA, and polymers.

To understand the emissive mechanism the XPS spectra of CNF and poly(TA) was determined. High resolution of O1s spectra indicated that the CNF had both hydroxyl and carboxylic moieties and poly(TA) had carboxylic moieties (**Figure** **4**a).^[^ ^63^, ^64^ ^]^ The interaction between poly(TA) and CNF was verified by FT‐IR. Poly(TA)/Cell exhibited signals at 1699 cm^−1^ for C═O and 3331 cm^−1^ for ─O─H. While, poly(TA), exhibited signals at 1701 cm^−1^ for C═O, and CNF exhibited signals at 3345 cm^−1^ for ─O─H, respectively (Figure 4b). Probably because in such molecules both the shortening and lengthening effects can be balanced,^[^ ^65^ ^]^ so hydrogen bond formation results in a slight red shift. Such redshifted signals confirmed the formation of hydrogen bonding interactions between poly(TA) and CNF. Theoretical simulations further confirmed the hydrogen bonding interaction between CNF and poly(TA) in Poly(TA)/Cell (Figure 4c). Specifically, the calculation shows three possible interaction scenarios formed in Poly(TA)/Cell: i. the carboxyl group of poly(TA) with the carboxyl group of CNF; ii. the carboxyl group of poly(TA) with the hydroxyl group of CNF; iii. the carboxyl group of poly(TA) with both the carboxyl and hydroxyl groups of CNF. Among these scenarios, the third interaction has the highest binding energy of −28.3 kcal mol^−1^ (Figure 4d; Figure S16, Supporting Information). The strong intermolecular interaction between CNF and poly(TA) is beneficial for the formation of multiple molecular clusters between these oxygen‐incorporated units. The as‐formed multiple clusters resulted in excitation‐dependent RTP emission via through‐space conjugation effect, which is illustrated in Figure 4c.

Having determined the origin of the RTP, the correlation between structure and performance of poly(TA) and CNF in Poly(TA)/Cell was further evaluated. To verify the role of poly(TA), a control sample, Poly(AA)/Cell, was prepared via physically mixing poly acrylic acid and CNF. Previous research had indicated that the carboxylic moieties exhibit the strongest interactions between poly(TA) and CNF, which results in RTP emission. However, although poly(AA) also possessed abundant carboxylic groups the as‐obtained Poly(AA)/Cell exhibited short lifetimes (Figure 4e). This suggests that the sulfur atoms,^[^ ^66^ ^]^ enhance the spin‐orbit coupling of the clusters in the Poly(TA)/Cell, which also contributes to the RTP emission.

Additionally, a control sample, Poly(TA)/PVA, was prepared via physically mixing poly thioctic acid and poly vinyl alcohol. In this composite, PVA, which shared similar abundant hydroxyl moieties as CNF. However, Poly(TA)/PVA exhibits short RTP lifetime of 30.0 ms (Figure 4e). Comparison of XRD spectra between CNF and PVA provides some clues for the reason. Specifically, XRD indicates that CNF exhibits a higher crystalline structure than PVA, which is particularly beneficial for providing a rigid environment and is favorable for RTP emission (Figure 4f).

2D correlation spectroscopy (2DCOS) was further employed to extract more subtle information about interaction changes within the Poly(TA)/Cell (Figure S17, Supporting Information). Synchronous and asynchronous spectra were generated to reflect synchronized and unsynchronized changes in spectral intensities at specific wavenumbers, respectively. Based on Noda's judging rule (see determination details in Table S1, Supporting Information),^[^ ^67^, ^68^, ^69^, ^70^ ^]^ the responsive order of different C═O groups to heat was determined as follows: 1575 cm^−1^ → 1675 cm^−1^ → 1709 cm^−1^ → 1610 cm^−1^ (→ means prior to or earlier than), corresponding to *v*(COOH) (free, CNF) → *v*(COOH) (free, Poly(TA)) → *v*(COO^−^) (hydrogen bond, Poly(TA)) → *v*(COO^−^) (hydrogen bond, CNF). As such the molecular clusters formed between CNF and poly(TA) via hydrogen bonding interactions, enhanced SOC assisted by sulfur atoms in poly(TA) and rigid environment facilitated by crystalline CNF, collectively contribute to the RTP emission of Poly(TA)/Cell.

### 2.4 Processability, Applications, and Recyclability

**Figure 5:**
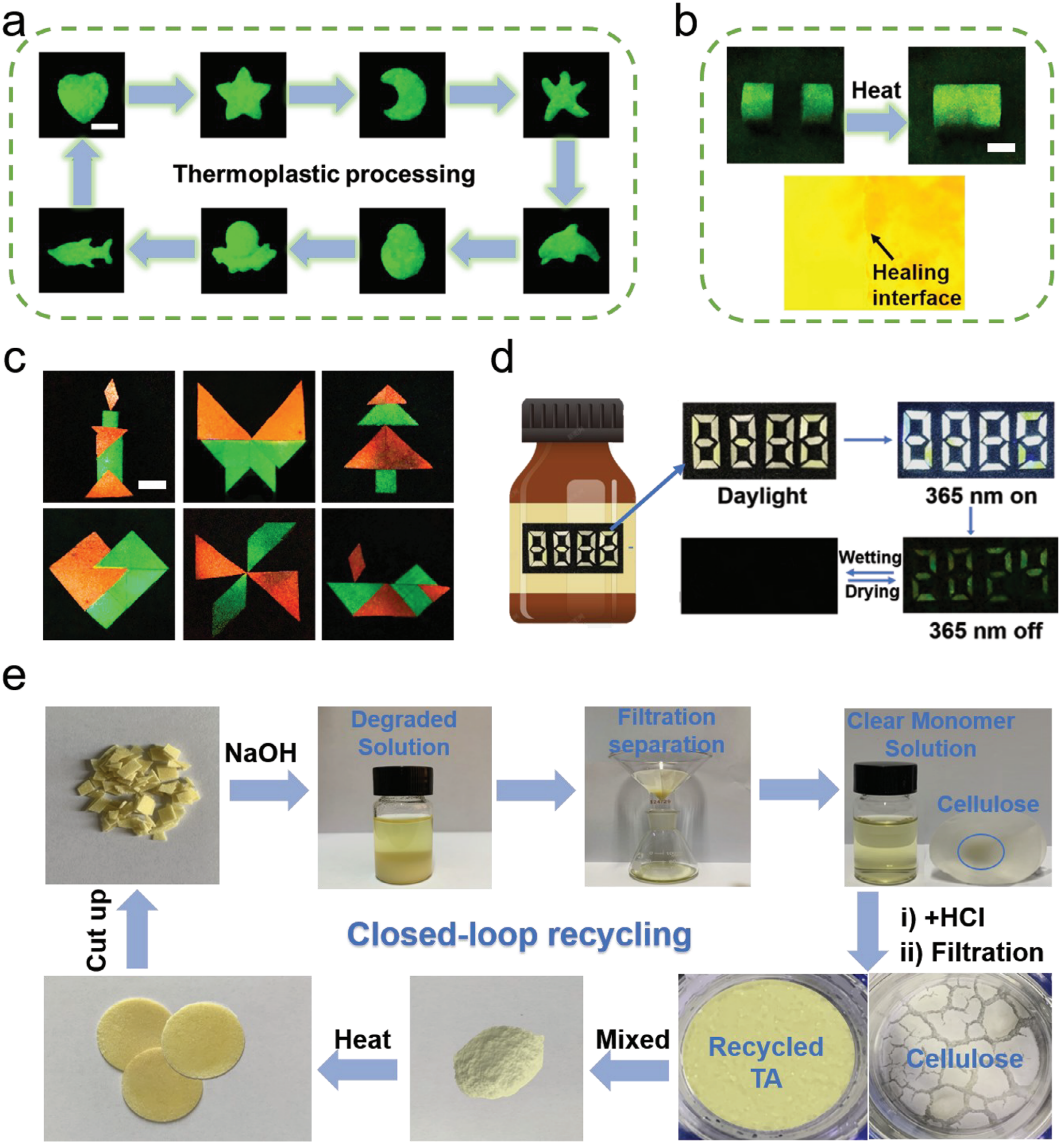
Applications and recyclability. a) The images of thermoplastic processability made from Poly(TA)/Cell. b) Photographs of self‐healing properties of Poly(TA)/Cell. c) The images of assembly of 2D materials made from Poly(TA)/Cell and Poly(TA)/Cell/RhB after removing the UV light source, scale bar = 1 cm. d) Smart anti‐counterfeiting logo for a medicine bottle made from different phosphors in the bright field, UV field and after removing the UV light source. e) Photographs of the facile purification procedure of TA monomers from the degraded mixture.

Taking advantages of the thermoplastic processability, Poly(TA)/Cell could be processed into flexible shapes using heating (80 °C) and cooling treatments (**Figure** **5**a). Notably, the RTP lifetime was not obviously compromised after thermal setting (Figure S18, Supporting Information). Moreover, the thermoplastic processing samples did not show any lifetime decrease after storage for a month (Figure S19, Supporting Information).

More interestingly, this Poly(TA)/Cell exhibits a self‐healing effect. Under low‐temperature heating conditions, the material can self‐repair, which is attributed to the dynamic covalent disulfide bonds of thioctic acid (Figure 5b). The tensile stress‐strain curves showed that Poly(TA)/Cell did not show any tensile strength decrease significantly after self‐healing (Figure S20, Supporting Information). Using these properties, the Poly(TA)/Cell and Poly(TA)/Cell/RhB was assembled by heating to create 2D pattern puzzles for decorations (Figure 5c).

Furthermore, an anti‐counterfeiting label for a medicine bottle was created using Poly(TA)/Cell (Figure 5d). The digital pattern “8888” was generated using prepared Poly(TA)/Cell in different ratios, specifically 1:0.1 and 1:1. Under excitation using a UV lamp, this pattern displays yellow–white fluorescence, and the pattern “8888” in yellow–white. Upon removal of the UV light source, an encrypted numeric pattern “2024” appears in yellow‐green phosphorescence (RTP), easily detectable by the naked eye.

Molecular recyclability of Poly(TA)/Cell was also demonstrated (Figure 5e). Using the base‐triggered cleavage of the disulfide bonds, Poly(TA)/Cell was dissolved in dilute alkaline solution for depolymerization. The as‐obtained depolymerized products consisted of water‐soluble deprotonated TA monomers and CNF precipitates, which were then separated by filtration. Specifically, the transparent filtrate containing TA monomers and excess alkali is acidified with 1 m hydrochloric acid (pH 3–4), protonating the TA monomers and precipitating them as a yellow powder. The CNF is then washed multiple times with water to remove the alkali, followed by vacuum drying. ^1^H NMR analysis confirmed that the recovered TA exhibit signals identical to the original materials (Figure S21, Supporting Information). The recovery rates of thioctic acid and CNF used for preparing Poly(TA)/Cell were ≈92.3% and 81.5%, respectively.

## 3 Conclusion

In summary, we have developed bio‐based thermoplastic Poly(TA)/Cell with closed‐loop recyclability using CNF and thioctic acid. Attributed to the multiple hydrogen bonding interactions between CNF and poly(TA), Poly(TA)/Cell exhibited excitation and humidity‐sensitive green RTP emission. Using energy transfer between Poly(TA)/Cell and RhB, by loading RhB into Poly(TA)/Cell red afterglow emissions were observed. The as‐obtained Poly(TA)/Cell could be thermally molded into flexible shapes with uncompromised RTP performance. Moreover, the Poly(TA)/Cell could self‐repair and the monomers used for generating Poly(TA)/Cell could be efficiently recycled, as a result of the dynamic and cleavable properties of the disulfide bonds in Poly(TA)/Cell. As a proof of applicability demonstration, Poly(TA)/Cell was used to create visual decorations and anti‐counterfeiting materials. We anticipate that this research will encourage and promote the practical, large‐scale and widespread application of sustainable RTP materials.

## 4 Experimental Section

### Preparation of Poly(TA)/Cell

Thioctic acid (TA) and cellulose (carboxylated cellulose nanofibers) were physically mixed at specific ratios, and the powder was heated with stirring in a container. After cooling to room temperature, Poly(TA)/Cell was obtained.

### Preparation of Poly(TA)/Cell /RhB

A certain amount of RhB was weighed and dissolved in water (20 mL) to prepare aqueous solutions of RhB. Poly(TA)/Cell was directly immersed in the different concentrations of RhB as aqueous solutions for 8 h at room temperature. After that, the sample was dried at 60 °C for 3.5 h to provide Poly(TA)/Cell/RhB. The loading capacity of Poly(TA)/Cell for RhB was calculated by measuring the increase in weight.

### Preparation of Poly(TA)/Cell‐M

The Poly(TA)/Cell (1.0 cm × 1.0 cm) was treated with 0.2 mol·L^−1^ CaCl_2_, CrCl_3_, MnCl_2_, FeCl_3_, CoCl_2_, HgCl_2_, PbCl_2_, CuCl_2_, and ZnCl_2_ aqueous solutions for 1 h, respectively.

## Conflict of Interest

The authors declare no conflict of interest.

## Supporting information



Supporting Information

## Data Availability

The authors declare that the main data are available in this article and its supplementary information files. Extra data are available from the corresponding author on reasonable request.

## References

[1] M. Vasilopoulou , A. R. bin Mohd Yusoff , M. Daboczi , J. Conforto , A. E. X. Gavim , W. J. da Silva , A. G. Macedo , A. Soultati , G. Pistolis , F. K. Schneider , Y. Dong , P. Jacoutot , G. Rotas , J. Jang , G. C. Vougioukalakis , C. L. Chochos , J. S. Kim , N. Gasparini , Nat. Commun. 2021, 12, 4868. 34381038 10.1038/s41467-021-25135-z PMC8357948

[2] J. Sun , H. Ahn , S. Kang , S. Ko , D. Song , H. A. Um , S. Kim , Y. Lee , P. Jeon , S. Hwang , Y. You , C. Chu , S. Kim , Nat. Photonics 2022, 16, 212.

[3] Y. Wang , H. Gao , J. Yang , M. Fang , D. Ding , B. Z. Tang , Z. Li , Adv. Mater. 2021, 33, 2007811. 10.1002/adma.202007811 33772942

[4] X.‐Y. Dai , M. Huo , X. Dong , Y.‐Y. Hu , Y. Liu , Adv. Mater. 2022, 34, 2203534. 10.1002/adma.202203534 35771589

[5] X. Zhang , J. Liu , B. Chen , X. He , X. Li , P. Wei , P. F. Gao , G. Zhang , J. W. Y. Lam , B. Z. Tang , Matter 2022, 5, 3499.

[6] N. Gan , X. Zou , M. Dong , Y. Wang , X. Wang , A. Lv , Z. Song , Y. Zhang , W. Gong , Z. Zhao , Z. Wang , Z. Zhou , H. Ma , X. Liu , Q. Chen , H. Shi , H. Yang , L. Gu , Z. An , W. Huang , Nat. Commun. 2022, 13, 3995. 35810179 10.1038/s41467-022-31554-3 PMC9271082

[7] X. Wang , W. Sun , H. Shi , H. Ma , G. Niu , Y. Li , J. Zhi , X. Yao , Z. Song , L. Chen , S. Li , G. Yang , Z. Zhou , Y. He , S. Qu , M. Wu , Z. Zhao , C. Yin , C. Lin , J. Gao , Q. Li , X. Zhen , L. Li , X. Chen , X. Liu , Z. An , H. Chen , W. Huang , Nat. Commun. 2022, 13, 5091. 36042210 10.1038/s41467-022-32054-0 PMC9428140

[8] J. Ma , F. Zeng , X. Lin , Y. Wang , Y. Ma , X. Jia , J. Zhang , B. Liu , Y. Wang , H. Zhao , Science 2024, 385, 68. 38963855 10.1126/science.adn5694

[9] C. Wang , L. Qu , X. Chen , Q. Zhou , Y. Yang , Y. Zheng , X. Zheng , L. Gao , J. Hao , L. Zhu , B. Pi , C. Yang , Adv. Mater. 2022, 34, 2204415. 10.1002/adma.202204415 35731029

[10] Z. Wang , Y. Zhang , C. Wang , X. Zheng , Y. Zheng , L. Gao , C. Yang , Y. Li , L. Qu , Y. Zhao , Adv. Mater. 2020, 32, 1907355. 10.1002/adma.201907355 31930607

[11] Y. Gao , W. Ye , K. Qiu , X. Zheng , S. Yan , Z. Wang , Z. An , H. Shi , W. Huang , Adv. Mater. 2023, 35, 2306501. 10.1002/adma.202306501 37793797

[12] S. Xiong , Y. Xiong , D. Wang , Y. Pan , K. Chen , Z. Zhao , D. Wang , B. Z. Tang , Adv. Mater. 2023, 35, 2301874. 10.1002/adma.202301874 37026437

[13] W. Zhao , Z. He , B. Z. Tang , Nat. Rev. Mater. 2020, 5, 869.

[14] X. Dai , M. Huo , Y. Liu , Nat. Rev. Chem. 2023, 7, 854. 37993737 10.1038/s41570-023-00555-1

[15] G. Baryshnikov , B. Minaev , H. Agren , Chem. Rev. 2017, 117, 6500. 28388041 10.1021/acs.chemrev.7b00060

[16] Z. Cheng , H. Shi , H. Ma , L. Bian , Q. Wu , L. Gu , S. Cai , X. Wang , W. Xiong , Z. An , W. Huang , Angew. Chem. Int. Ed. 2018, 57, 678. 10.1002/anie.201710017 29205713

[17] J. Song , Y. Zhou , Z. Pan , Y. Hu , Z. He , H. Tian , X. Ma , Matter 2023, 6, 2005.

[18] Z. Pan , J. Song , S. Zhang , P. Zeng , J. Mei , D. Qu , Sci. Bull. 2024, 69, 1237. 10.1016/j.scib.2024.02.029 38458915

[19] Y. Hu , X. Dai , X. Dong , M. Huo , Y. Liu , Angew. Chem. Int. Ed. 2022, 61, e202213097. 10.1002/anie.202213097 36094757

[20] W. Zhou , Y. Chen , Q. Yu , H. Zhang , Z. Liu , X. Dai , J. Li , Y. Liu , Nat. Commun. 2020, 11, 4655. 32938918 10.1038/s41467-020-18520-7 PMC7494876

[21] Y. Zhang , Y. Su , H. Wu , Z. Wang , C. Wang , Y. Zheng , X. Zheng , L. Gao , Q. Zhou , Y. Yang , X. Chen , C. Yang , Y. Zhao , J. Am. Chem. Soc. 2021, 143, 13675. 34410121 10.1021/jacs.1c05213

[22] Y. Yang , A. Li , Y. Yang , J. Wang , Y. Chen , K. Yang , B. Z. Tang , Z. Li , Angew. Chem. Int. Ed. 2023, 62, e202308848. 10.1002/anie.202308848 37590031

[23] Z. Zhao , P. Zhao , S. Chen , Y. Zheng , J. Zuo , C. Li , Angew. Chem. Int. Ed. 2023, 62, e202301993. 10.1002/anie.202301993 36995342

[24] E. Hamzehpoor , C. Ruchlin , Y. Tao , C. Liu , H. M. Titi , D. F. Perepichka , Nat. Chem. 2023, 15, 83. 36302870 10.1038/s41557-022-01070-4

[25] S. Xu , Q. Zhang , Mater. Today Energy 2021, 20, 100635.

[26] B. Zhou , Z. Qi , D. Yan , Angew. Chem. Int. Ed. 2022, 61, e202208735. 10.1002/anie.202208735 35819048

[27] X. Yang , D. Yan , Chem. Sci. 2016, 7, 4519. 30155098 10.1039/c6sc00563b PMC6016333

[28] Q. Xia , J. Yu , Z. Chen , Z. Xue , X. Wang , X. Liu , M. Wu , Cell Rep. Phys. Sci. 2023, 4, 101494.

[29] X. Xu , B. Yan , Adv. Opt. Mater. 2022, 10, 2200451.

[30] X. Luo , B. Tian , Y. Zhai , H. Guo , S. Liu , J. Li , S. Li , T. D. James , Z. Chen , Nat. Rev. Chem. 2023, 7, 800. 37749285 10.1038/s41570-023-00536-4

[31] B. Y. Karlinskii , V. P. P. Ananikov , Chem. Soc. Rev. 2023, 52, 836. 36562482 10.1039/d2cs00773h

[32] K. Wan , B. Tian , Y. Zhai , Y. Liu , H. Wang , S. Liu , S. Li , W. Ye , Z. An , C. Li , J. Li , T. D. James , Z. Chen , Nat. Commun. 2022, 13, 5508. 36127373 10.1038/s41467-022-33273-1 PMC9489714

[33] Y. Zhai , S. Li , J. Li , S. Liu , T. D. James , J. L. Sessler , Z. Chen , Nat. Commun. 2023, 14, 2614. 37147300 10.1038/s41467-023-37762-9 PMC10162966

[34] J. You , X. Zhang , Q. Nan , K. Jin , J. Zhang , Y. Wang , C. Yin , Z. Yang , J. Zhang , Nat. Commun. 2023, 14, 4163. 37443312 10.1038/s41467-023-39767-w PMC10344924

[35] X. Nie , J. Gong , Z. Ding , B. Wu , W.‐J. Wang , F. Gao , G. Zhang , P. Alam , Y. Xiong , Z. Zhao , Z. Qiu , B. Z. Tang , Adv. Sci. 2024, 11, 2405327. 10.1002/advs.202405327 PMC11434032 38952072

[36] B. Lu , Q. Gao , P. Li , J. Rao , Z. Lv , M. Shi , Y. Hu , X. Hao , G. Chen , M. Yin , F. Peng , Cell Rep. Phys. Sci. 2022, 3, 101015.

[37] M. Shi , Q. Gao , J. Rao , Z. Lv , M. Chen , G. Chen , J. Bian , J. Ren , B. Lu , F. Peng , J. Am. Chem. Soc. 2023, 146, 1294. 38054299 10.1021/jacs.3c07034

[38] J. Zhou , B. Tian , Y. Zhai , M. Wang , S. Liu , J. Li , S. Li , T. D. James , Z. Chen , Nat. Commun. 2024, 15, 7198. 39169019 10.1038/s41467-024-51545-w PMC11339440

[39] R. Liu , T. Jiang , D. Liu , X. Ma , Sci. China Chem. 2022, 65, 1100.

[40] S. Cai , Z. Sun , H. Wang , X. Yao , H. Ma , W. Jia , S. Wang , Z. Li , H. Shi , Z. An , Y. Ishida , T. Aida , W. Huang , J. Am. Chem. Soc. 2021, 143, 16256. 34550674 10.1021/jacs.1c07674

[41] K. Wan , Y. Zhai , S. Liu , J. Li , S. Li , B. Strehmel , Z. Chen , T. D. James , Angew. Chem. Int. Ed. 2022, 61, e202202760. 10.1002/anie.202202760 35388962

[42] M. Chen , C. Liu , H. Sun , F. Yang , D. Hou , Y. Zheng , R. Shi , X. He , X. Lin , ACS Appl. Mater. Interfaces 2024, 16, 9182. 38343193 10.1021/acsami.3c18131

[43] X. Zhang , Y. Cheng , J. You , J. Zhang , C. Yin , J. Zhang , Nat. Commun. 2022, 13, 1117. 35236853 10.1038/s41467-022-28759-x PMC8891296

[44] M. Zhou , D. Chen , Q. Chen , P. Chen , G. Song , C. Chang , Adv. Mater. 2024, 36, 2312220. 10.1002/adma.202312220 38288877

[45] M. Zeng , T. Li , Y. Liu , X. Lin , X. Zu , Y. Mu , L. Chen , Y. Huo , Y. Qin , Chem. Eng. J. 2022, 446, 136935.

[46] C. Shi , D. He , Q. Zhang , F. Tong , Z. Shi , H. Tian , D. Qu , Natl. Sci. Rev. 2023, 10, nwac139. 36994382 10.1093/nsr/nwac139 PMC10042223

[47] C. Shi , D. He , B. Wang , Q. Zhang , H. Tian , D. Qu , Angew. Chem. Int. Ed. 2023, 62, e202214422. 10.1002/anie.202214422 36378119

[48] C. Shi , Q. Zhang , B. Wang , D. He , H. Tian , D. Qu , CCS Chem. 2023, 5, 1422.

[49] H. Qiao , B. Wu , S. Sun , P. Wu , J. Am. Chem. Soc. 2024, 146, 7533. 38451015 10.1021/jacs.3c13392

[50] X. Jiang , Y. Hou , Y. Wang , F. Chu , J. Zhu , L. Song , Y. Hu , W. Hu , Adv. Funct. Mater. 2024, 2412313.

[51] C. He , F. Liang , L. Veeramuthu , C. Cho , J. Benas , Y. Tzeng , Y. Tseng , W. Chen , A. Rwei , C. Kuo , Adv. Sci. 2021, 8, 2102275. 10.1002/advs.202102275 PMC8564429 34519441

[52] T. Sun , M. Venkatesan , Y. Hsu , J. Chandrasekar , W. Chen , J. Benas , C. Cho , J. Lin , F. Liang , A. Y. Rwei , C. Kuo , Nano Energy 2025, 133, 110416.

[53] F. Liang , F. Jhuang , Y. Fang , J. Benas , W. Chen , Z. Yan , W. Lin , C. Su , Y. Sato , T. Chiba , J. Kido , C. Kuo , Adv. Mater. 2023, 35, 2207617. 10.1002/adma.202207617 36353914

[54] X. Yang , B. Zhang , J. Li , M. Shen , H. Liu , X. Xu , S. Shang , Carbohydr. Polym. 2023, 313, 120813. 37182943 10.1016/j.carbpol.2023.120813

[55] C. Dang , Y. Shao , S. Ding , H. Qi , W. Zhai , Adv. Mater. 2024, 36, 2406967. 10.1002/adma.202406967 39248650

[56] Q. Zhang , C. Shi , D. Qu , Y. Long , B. L. Feringa , H. Tian , Sci. Adv. 2018, 4, eaat8192. 30062126 10.1126/sciadv.aat8192 PMC6063538

[57] K. Jin , G. Song , H. Diao , X. Zhang , X. Ji , J. Zhang , J. Zhang , Cellulose 2023, 30, 8139.

[58] Q. Zhang , Y. Deng , C. Shi , B. L. Feringa , H. Tian , D. Qu , Matter 2021, 4, 1352.

[59] M. Chen , R. Yang , H. Wu , Q. Wang , C. Shi , S. Zhou , D. Yang , F. Liu , H. Tian , D. Qu , Angew. Chem. Int. Ed. 2024, 163, e202409200. 10.1002/anie.202409200 39031788

[60] M. Xie , F. Xu , L. Zhang , J. Yin , X. Jianet , ACS Macro Lett. 2018, 7, 540. 35632928 10.1021/acsmacrolett.8b00211

[61] M. Bok , Z. Zhao , S. H. Hwang , J. Ahn , J. Ko , J.‐Y. Jung , J. Lee , S. Jeon , J.‐H. Jeong , ACS Nano 2022, 16, 18157. 36240045 10.1021/acsnano.2c05159

[62] S. Kuila , S. J. George , Angew. Chem. Int. Ed. 2020, 59, 9393. 10.1002/anie.202002555 32142188

[63] Q. Wang , D. Xie , J. Chen , G. Liu , M. Yu , J. Mater. Sci. 2020, 55, 7084.

[64] C. He , L. Ke , Y. Lei , W. Gong , H. Liang , Z. Xu , T. Li , S. Chen , D. Zhang , Eur. Polym. J. 2024, 212, 113051.

[65] J. Joseph , E. D. Jemmis , J. Am. Chem. Soc. 2007, 129, 4620. 17375920 10.1021/ja067545z

[66] L. Ma , X. Ma , Sci. China Chem. 2023, 66, 304.

[67] H. Ye , B. Wu , S. Sun , P. Wu , Nat. Commun. 2024, 15, 885. 38287011 10.1038/s41467-024-45079-4 PMC10825218

[68] W. Zhang , B. Wu , S. Sun , P. Wu , Nat. Commun. 2021, 12, 4082. 34215738 10.1038/s41467-021-24382-4 PMC8253733

[69] S. Sun , P. Wu , Chin. J. Polym. Sci. 2017, 35, 700.

[70] C. C. Hornat , M. W. Urban , Nat. Commun. 2020, 11, 1028. 32098954 10.1038/s41467-020-14911-y PMC7042321

