# Supplementary material for: Bio‐Based Thermoplastic Room Temperature Phosphorescent Materials with Closed‐Loop Recyclability
Source: Adv Sci (Weinh). 2025 Mar 14;12(17):2414439. doi: 10.1002/advs.202414439 (PMC12061272; doi:10.1002/advs.202414439)
Supplement: Supplementary file 1 — Supporting Information [file ADVS-12-2414439-s001.docx]

**Supporting Information**

**Bio-based** **thermoplastic room temperature phosphorescent materials with closed-loop** **recyclability**

*Yuanyuan Qian^#^, Yingxiang Zhai^#^, Meng Li*, Yinping Qin, Liang Lv, Tony D. James, Lidong Wang* and Zhijun Chen**

Y. Q, M. L, Y. Q, L. L, L. W

Hebei Key Lab of Power Plant Flue Gas Multi-Pollutants Control, Department of Environmental Science and Engineering, North China Electric Power University, Baoding, 071003, P. R. China

E-email: wld@ncepu.edu.cn; mlincepu@hotmail.com

Y. Z, Z. C

Key Laboratory of Bio-based Material Science & Technology, Northeast Forestry University, Harbin, 150040, P. R. China

E-email: chenzhijun@nefu.edu.cn

T. D. J

Department of Chemistry, University of Bath, Bath, BA2 7AY, UK

School of Chemistry and Chemical Engineering, Henan Normal University, Xinxiang, 453007, P. R. China

[*^#^*] These authors contributed equally to this work

**Materials.**

All reagents employed in this research were used without further purification. Carboxylated cellulose nanofibers (CNF-C), thioctic acid (TA), Rhodamine B (RhB), Copric chloride dihydrate (CuCl_2_·2H_2_O), Cobalt(II) chloride (CoCl_2_), Zinc chloride (ZnCl_2_), Chromium chloride hexahydrate (CrCl_3_·6H_2_O), Manganese chloride (MnCl_2_), Lead chloride (PbCl_2_), Calcium chloride anhydrous (CaCl_2_), Iron chloride hexahydrate (FeCl_3_·6H_2_O) and chromium chloride hexahydrate (CrCl_3_·6H_2_O) were purchased from Aladdin Chemical Reagent Co., Ltd. (Shanghai, China). Hydrochloric acid (HCl) was of analytical grade and were obtained from Tianjin Kermel Reagent Co., Ltd. Sodium hydroxide (NaOH) was purchased from Tianjin Damao Chemical Reagent Factory.

**Characterization.**

Fluorescence spectra, afterglow spectra, and lifetime decay curves were recorded using a FLS1000 photoluminescence spectrometer (Edinburgh Instruments, Livingston, UK) equipped with a xenon lamp and a one-microsecond lamp (detector: photoelectric multiplier, 200 nm < λ < 1700 nm). Afterglow emission spectra were recorded after a 10 ms delay. UV-Vis absorption spectra were recorded using a TU-1901 UV-Vis double-beam spectrophotometer (Persee General Instrument Co., Ltd., Beijing, China). Fourier transform infrared (FT-IR) spectra were obtained on Thermo Electron Nicolet iZ10 over the 4000-400 cm^-1^ spectral range. The morphology of the samples was evaluated using a scanning electron microscopy (SEM, Hitachi-s4800). The X-ray photoelectron spectrum (XPS) was performed to analyze the chemical composition of hydrogels using a Thermo Scientific: ESCALAB250Xi. X-ray diffraction experiments were undertaken on a rotating anode X-ray powder diffractometer (D8 ADVANCE, BRUKER). Dynamic mechanical analysis (DMA) tests were performed with a TA Q800 analyzer (TA Instruments, USA) in three-point bending mode with a frequency of 1 Hz and a heating rate of 5 °C min^−1^. Thermogravimetric analysis (TGA) was conducted with a thermogravimetric analyzer (TGA 4000, PerkinElmer) test. Sample was heated at a constant heating rate of 10 ℃/ min, from room temperature to 700 ℃, under inert (N_2_) atmosphere. 2DCOS analysis was carried out using the software, 2D Shige ver.1.3 (© Shigeaki Morita, Kwansei Gakuin University, Japan, 2004-2005) and further plotted into contour maps by OriginPro program. In the contour maps, red colors are defined as positive intensities, and blue colors are defined as negative ones. ^1^H NMR spectra were recorded on a Bruker AV-300 spectrometer with chemical shifts reported in ppm (in CDCl_3_) at room temperature.

**Simulation methods**

All calculations in this work were performed using Gaussian 16 program package^[1]^. Full geometry optimizations were performed to locate all the stationary points, using the B3LYP method with the def2svp basis^[2, 3]^, namely B3LYP/def2svp. Dispersion corrections were computed with Grimme's D3(BJ) method in optimization^[4]^. Harmonic vibrational frequency was performed at the same level to guarantee that there is no imaginary frequency in the molecules, i.e. they locate on the minima of potential energy surface. Convergence parameters of the default threshold were retained (maximum force within 4.5×10^−4^ Hartrees/Bohr and root mean square (RMS) force within 3.0×10^−4^ Hartrees/Radian) to obtain the optimized structure. The optimal structure was identified given that all calculations for structural optimization were successfully converged within the convergence threshold of no imaginary frequency, during the process of vibration analysis. The solvation energies are calculated by the literature^[5]^.

**Supplementary Figures:**

**
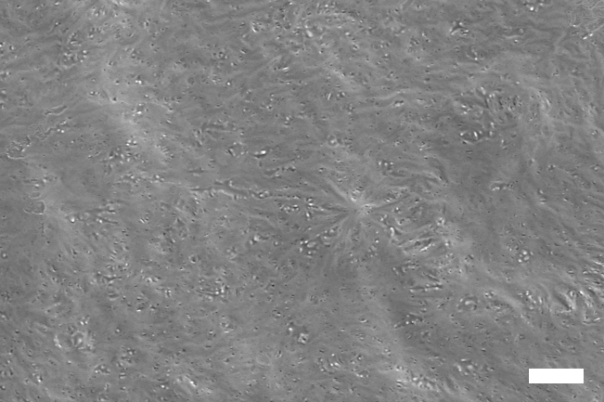
**

**Figure S1.** Scanning electron microscopic (SEM) images of Poly(TA)/Cell; scale bar = 50 μm.


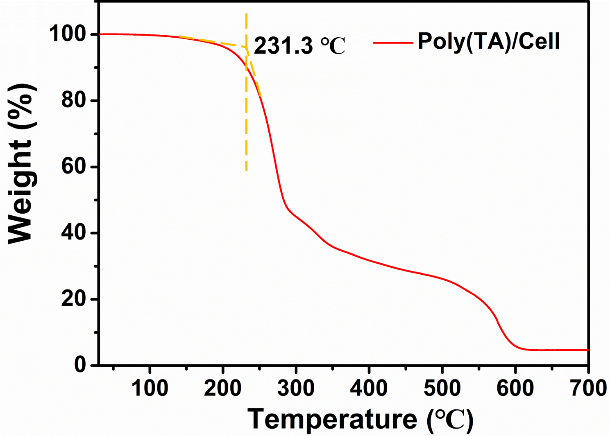


**Figure S2.** TGA heating curves of Poly(TA)/Cell.

**
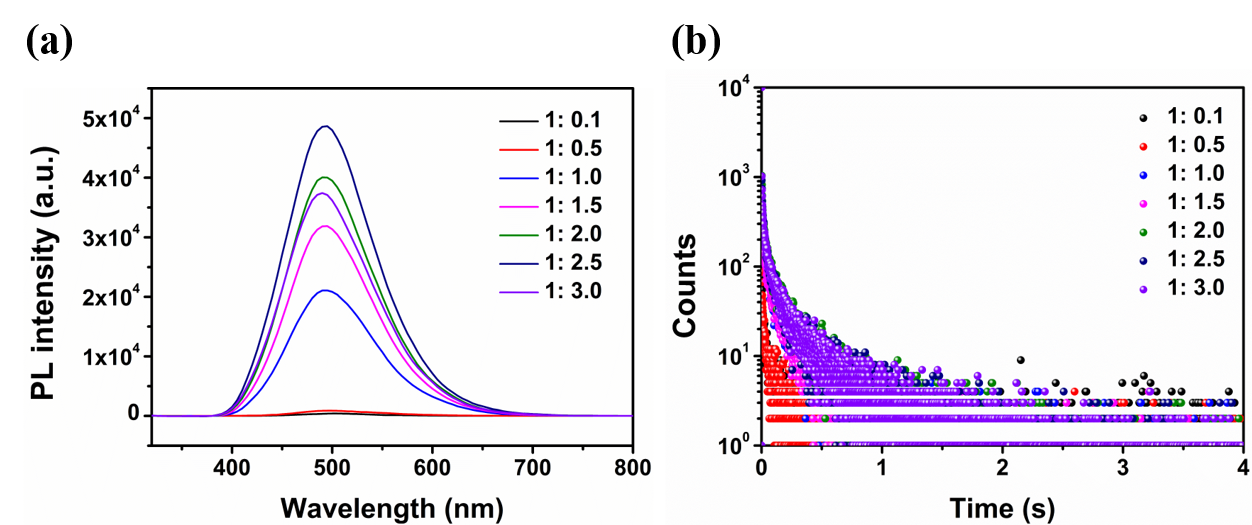
**

**Figure S3.** RTP emission spectra a) and lifetime b) of Poly(TA)/Cell with different CNF contents. Excitation wavelength = 300 nm.

**
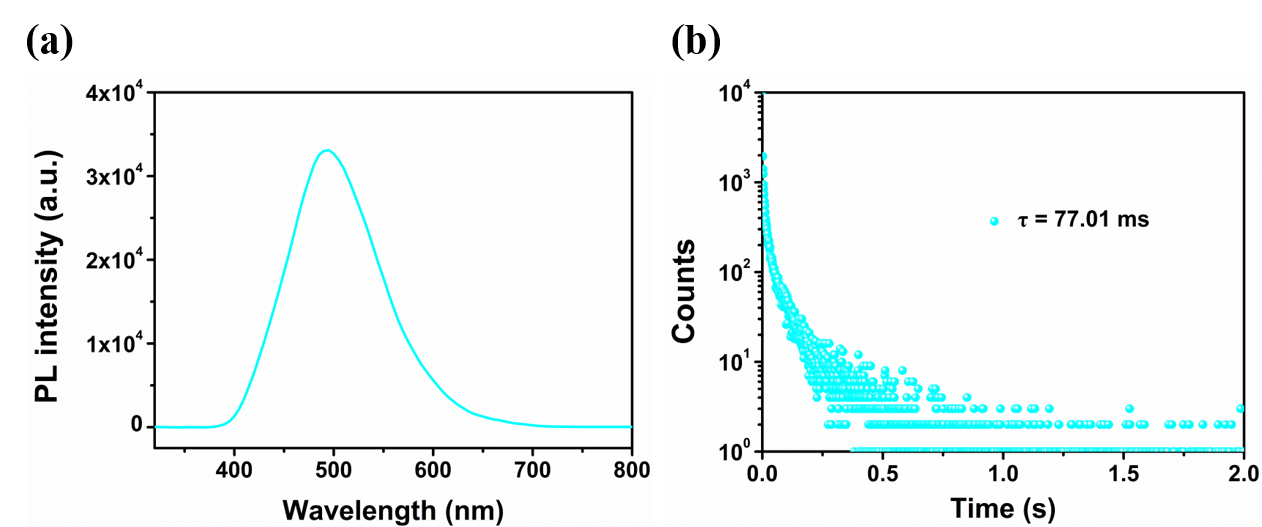
**

**Figure S4.** Afterglow emission spectra a) and lifetime b) of CNF. Excitation wavelength = 300 nm.


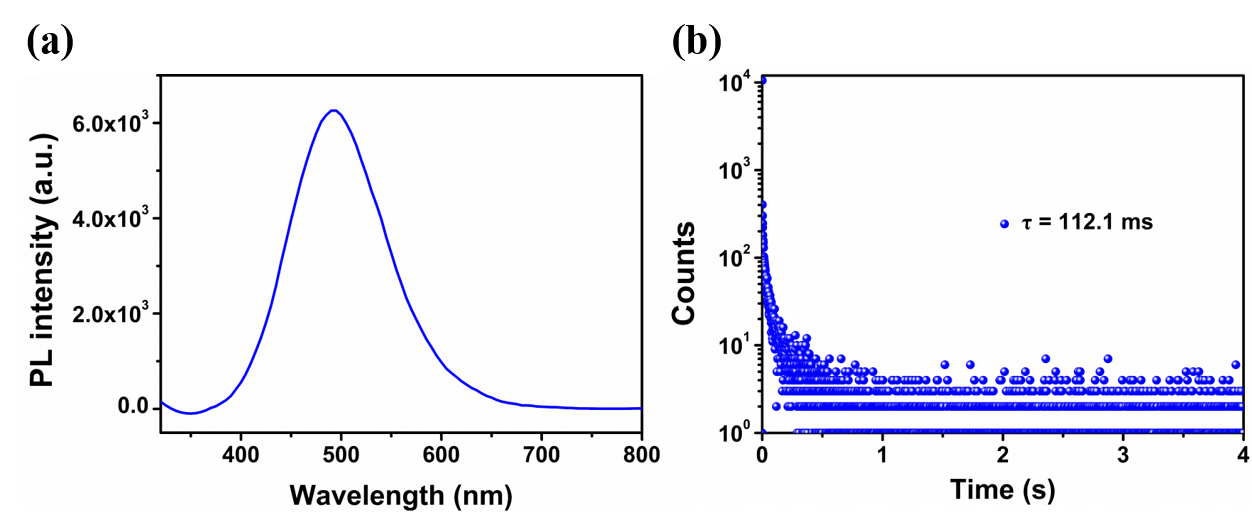


**Figure S5.** Afterglow emission spectra a) and lifetime b) of poly(TA). Excitation wavelength = 300 nm.

**

**

**Figure S6.** The phosphorescence lifetimes of the Poly(TA)/Cell in different excitation wavelengths.

**

**

**Figure S7.** RTP lifetime of Poly(TA)/Cell upon exposure to different humidity.

**
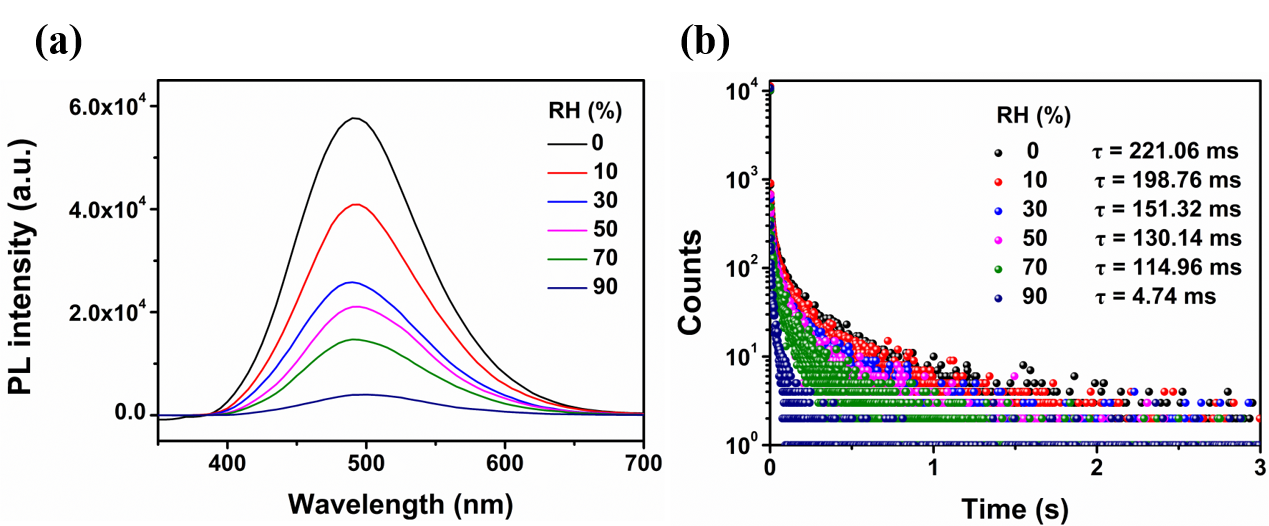
**

**Figure S8.** Phosphorescence properties of Poly(TA)/Cell under conditions of different relative humidity. a) Afterglow emission spectra of Poly(TA)/Cell determined under different relative humidity conditions. b) Lifetime decay profiles of Poly(TA)/Cell determined under different relative humidity conditions. Excitation wavelength = 300 nm.





**Figure S9.** RTP decay profiles of wood wax@Poly(TA)/Cell and wood wax@Poly(TA)/Cell treated with 90% relative humidity (RH). Excitation wavelength = 300 nm.


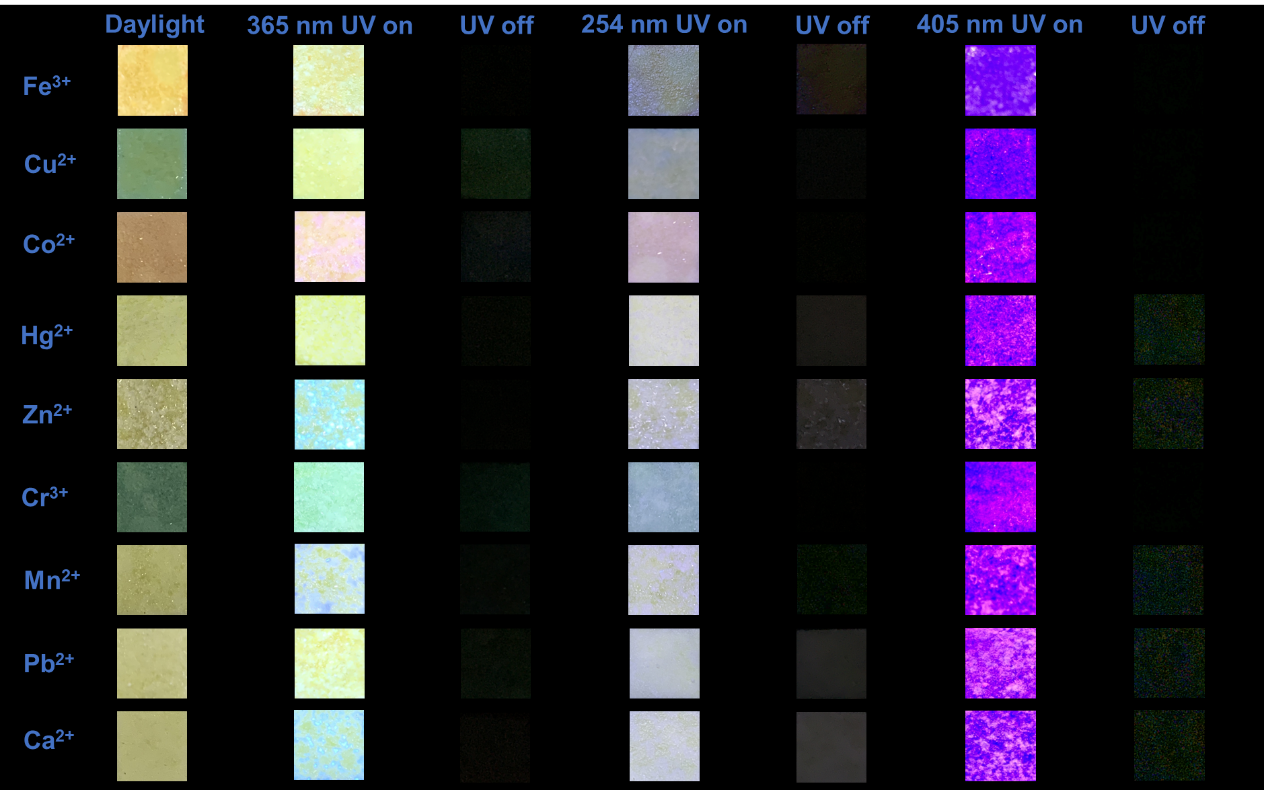


**Figure S10.** Photographs of the Poly(TA)/Cell treated with different metal cations under and after ceasing light irradiation.

**
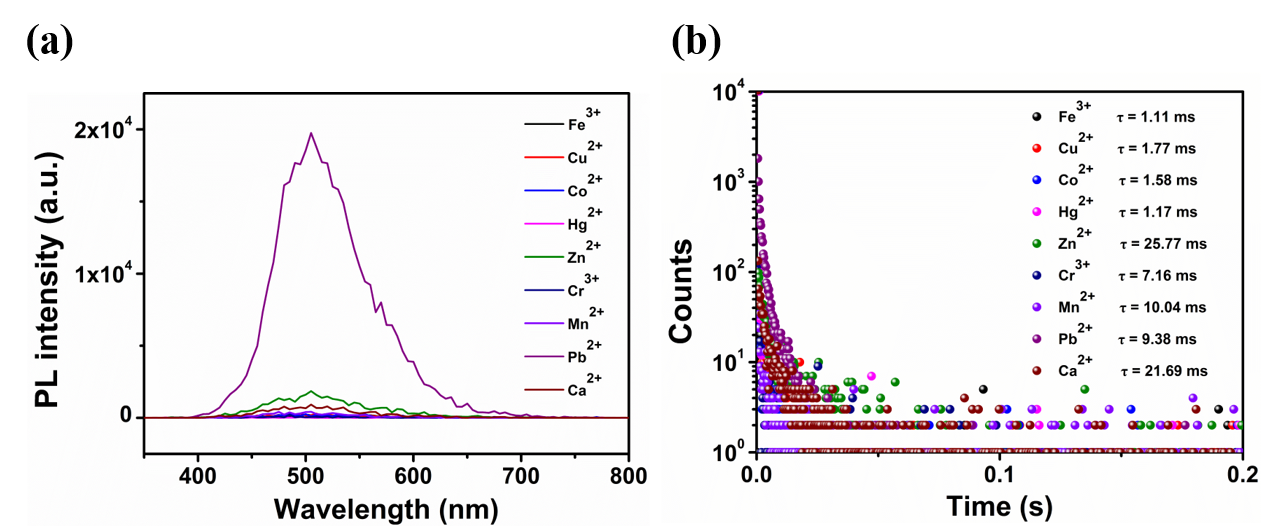
**

**Figure S11.** a) Afterglow emission spectra of Poly(TA)/Cell treated with different metal cations. b) Lifetime decay profiles of Poly(TA)/Cell treated with different metal cations. Excitation wavelength = 300 nm.

**

**

**Figure S12.** The RTP emission of Poly(TA)/Cell and the absorbance of RhB.

**

**

**Figure S13.** Lifetime decay of delayed fluorescence of Poly(TA)/Cell/RhB at 610 nm. Excitation wavelength = 300 nm.

**

**

**Figure S14.** Phosphorescence spectra of Poly(TA)/Cell/RhB at different RhB concentrations. Excitation wavelength = 300 nm.

**
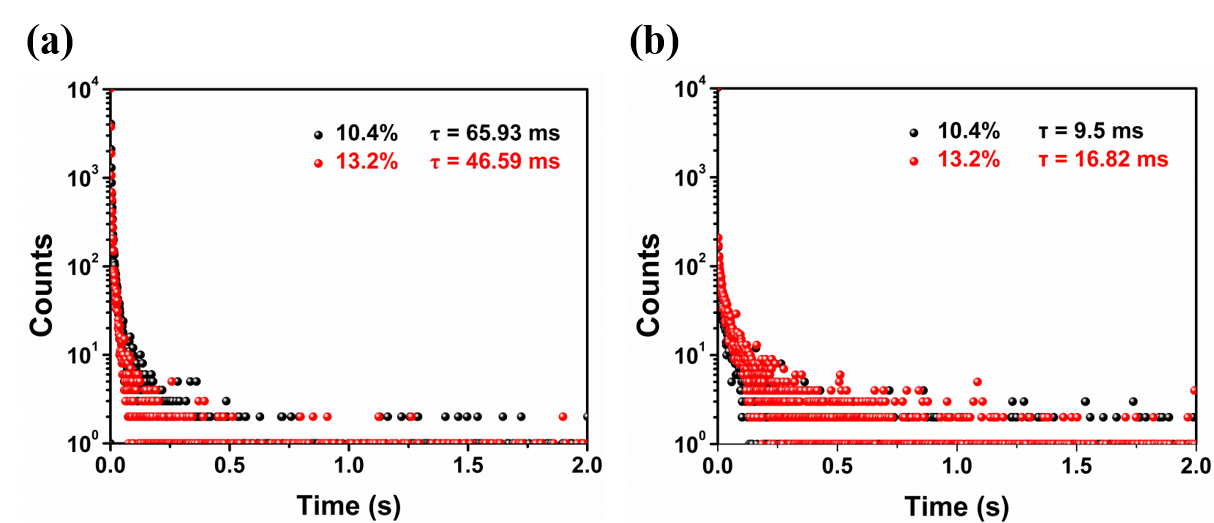
**

**Figure S15.** a) Lifetime of different RhB concentrations in Poly(TA)/Cell/RhB. Emission wavelength = 490 nm. b) Lifetime of different RhB concentrations in Poly(TA)/Cell/RhB. Emission wavelength = 610 nm.

**
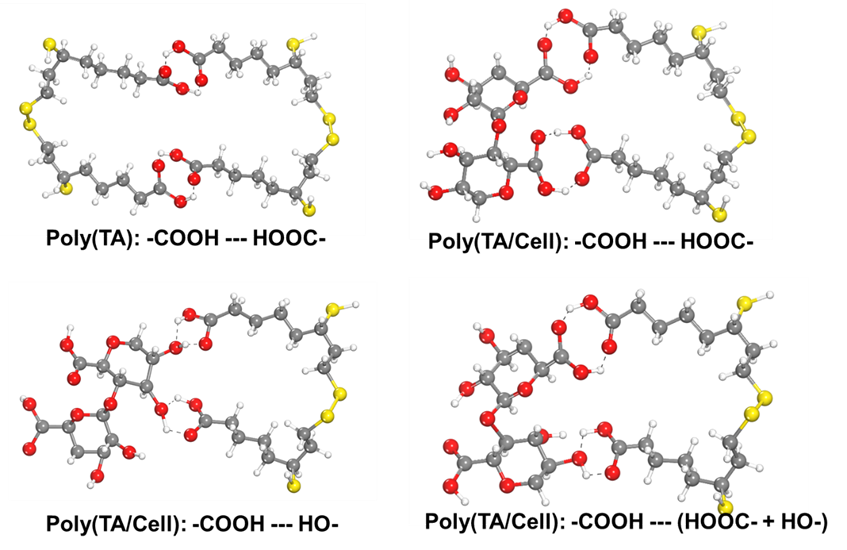
**

**Figure S16.** Simulation of interaction between thioctic acid/CNF.


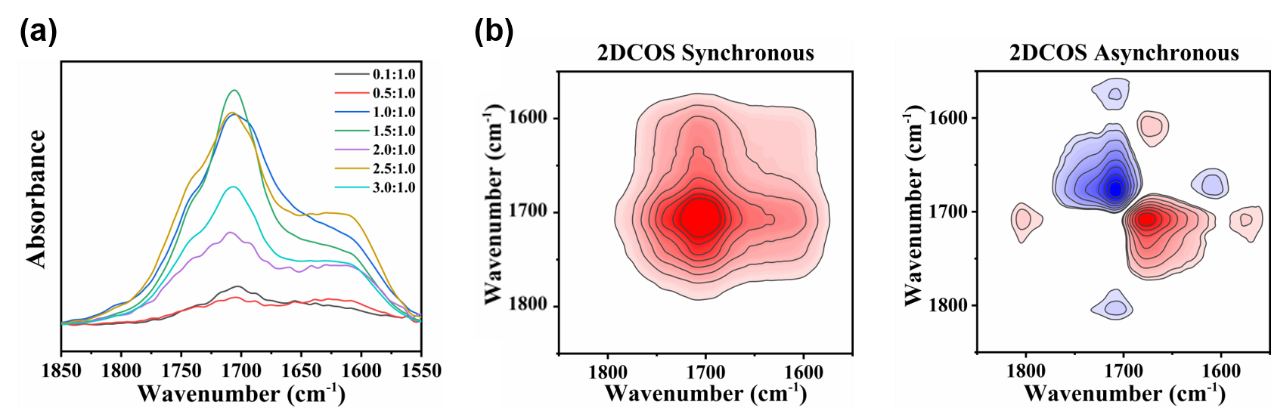


**Figure S17.** a) FTIR spectra of the Poly(TA)/Cell upon changing the mass ratio of CNF from 0.1 to 3. b) 2DCOS synchronous and asynchronous spectra generated from (a). In 2DCOS spectra, red colors represent positive intensities, while blue colors represent negative intensities.

**
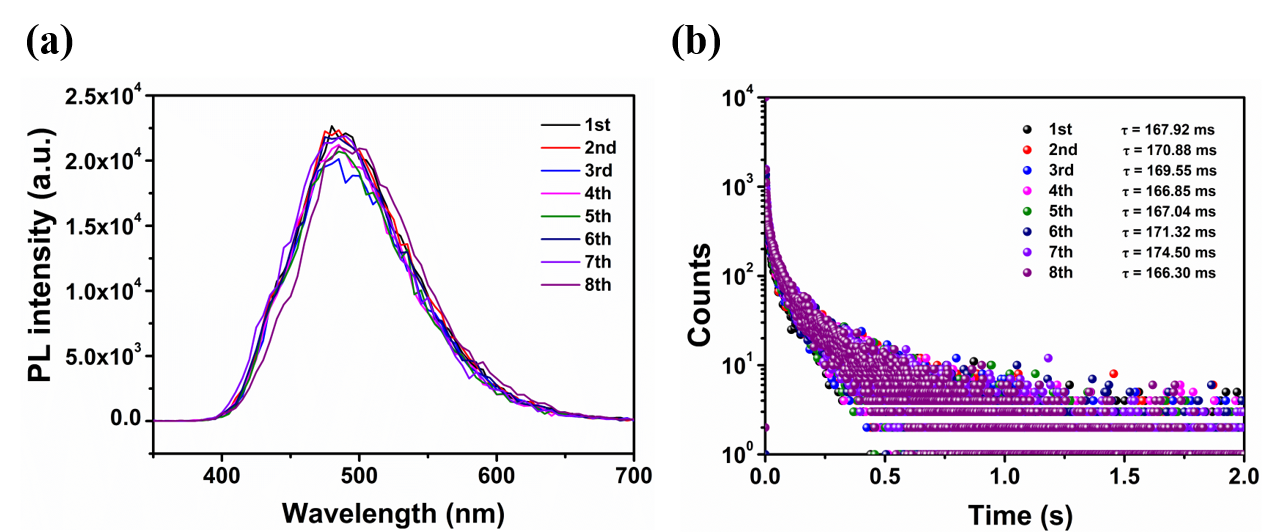
**

**Figure S18.** a) Afterglow emission spectra of Poly(TA)/Cell determined after thermal setting. b) Lifetime decay profiles of Poly(TA)/Cell determined after thermal setting. Excitation wavelength = 300 nm.

**

**

**Figure S19.** RTP decay profiles of Poly(TA)/Cell and Poly(TA)/Cell after storage for

a month. Excitation wavelength = 300 nm.





**Figure S20.** Stress-strain curves of the healed samples Poly(TA)/Cell with freshly cut interfaces.

**
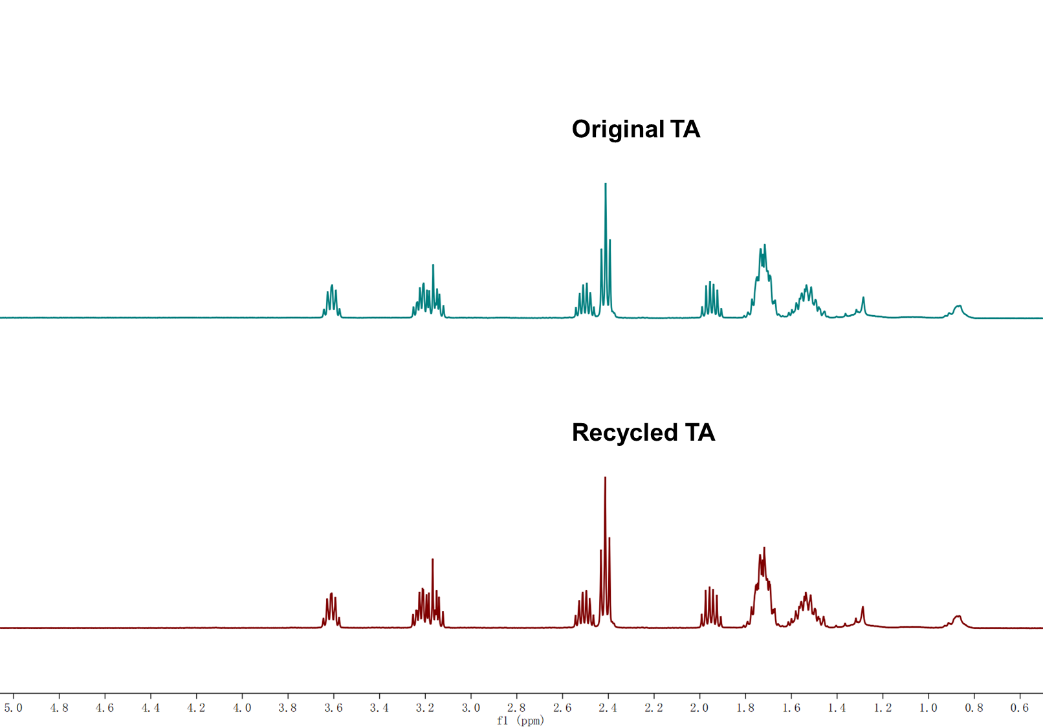
**

**Figure S21.** ^1^H NMR spectra of the original TA monomers and recycled monomers. (298K, CDCl_3_, 400 MHz).

**Table S1.** Summary of sign multiplication results for cross-peaks in 2DCOS synchronous and asynchronous spectra of the Poly(TA)/Cell.

| 1575 | - | - | - |  |
| --- | --- | --- | --- | --- |
| 1610 | + | + |  |  |
| 1675 | - |  |  |  |
| 1709 |  |  |  |  |
|  | 1709 | 1675 | 1610 | 1575 |

As reported in the literature, the sequence of spectral changes can be determined using Noda’s rule, which states that if the product of the signs of cross-peaks (*v*_1_, *v*_2_, and assume *v*_1_ > *v*_2_) in synchronous and asynchronous spectra is positive, the change at *v*_1_ precedes that at *v*_2_; otherwise, the change at *v*_2_ occurs prior to *v*_1_. In Table S1, “+” and “-” denote the same and different signs in synchronous and asynchronous spectra, respectively.

1. D. R. Meena, S. R. Gadre, P. Balanarayan, *Comput. Phys. Commun.* **2018**, *224*, 299-310.
2. F. Weigend, R. Ahlrichs, *Phys. Chem. Chem. Phys.* **2005**, *7*, 3297-3305.
3. S. Xu, T. He, J. Li, Z. Huang, C. Hu, *Appl. Catal., B* **2021**, *292*, 120145.
4. S. Grimme, J. Antony, S. Ehrlich, H. Krieg, *J. Chem. Phys.* **2010**, *132*, 154104.
5. R. Fang, H. Xu, B. Xu, X. Li, Y. Li, J. B. Goodenough, *Adv. Funct. Mater.* **2021**, *31*, 2001812.
